# Supplementary material for: A male-derived volatile sex pheromone in Caenorhabditis nematodes identified through its mimicry by a predator
Source: bioRxiv. 2025 Sep 17:2025.09.12.675966. Preprint. [Version 1] doi: 10.1101/2025.09.12.675966 (PMC12458352; doi:10.1101/2025.09.12.675966)
Supplement: Supplement 1 [file media-1.pdf]

**Supplementary Information**

# A male-derived volatile sex pheromone in *Caenorhabditis* nematodes identified through its mimicry by a predator

**Authors:** Matthew R. Gronquist[1], Xuan Wan[2], Daniel Leighton[3], Yuki Togawa[4], Marika Sagawa[4], Paul W. Sternberg[2]*, Frank C. Schroeder[5], Ryoji Shinya[4]*

**Affiliations:**

1. Department of Chemistry and Biochemistry, State University of New York at Fredonia, USA
2. Division of Biology and Biological Engineering, California Institute of Technology, USA
3. Current address: Department of Human Genetics, Department of Biological Chemistry, and Howard Hughes Medical Institute, University of California, Los Angeles, USA
4. School of Agriculture, Meiji University, Japan
5. Boyce Thompson Institute and Department of Chemistry and Chemical Biology, Cornell University, USA

*Corresponding authors

**Emails**

Paul Sternberg; pws@caltech.edu; 0000-0002-7699-0173

Ryoji Shinya; shinya@meiji.ac.jp; 0000-0002-2450-3045

# Supplementary Figures

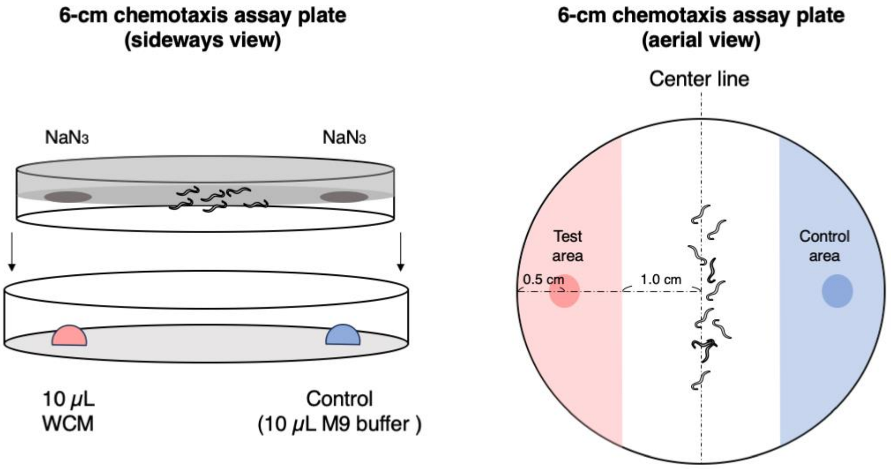


**Fig. S1: Schematic diagram of chemotaxis assay with worm conditioned media (WCM) on the 6-cm chemotaxis assay plate.**

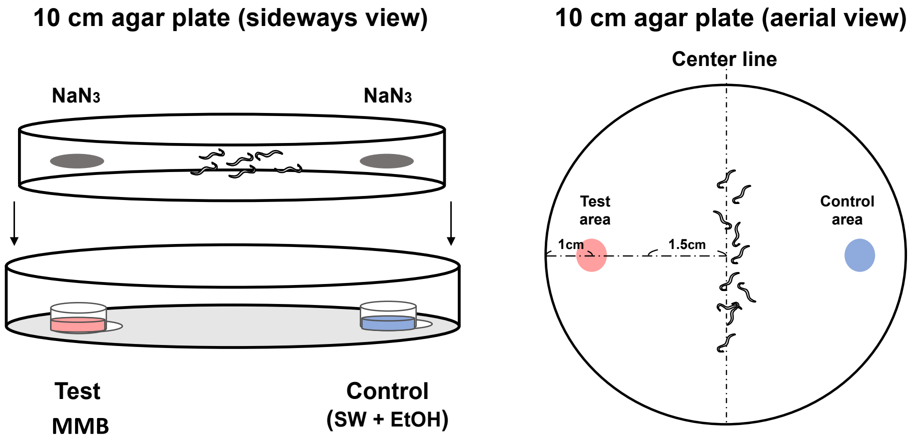


**Fig. S2: Schematic diagram of chemotaxis assay with MMB on the 10-cm chemotaxis assay plate. .**

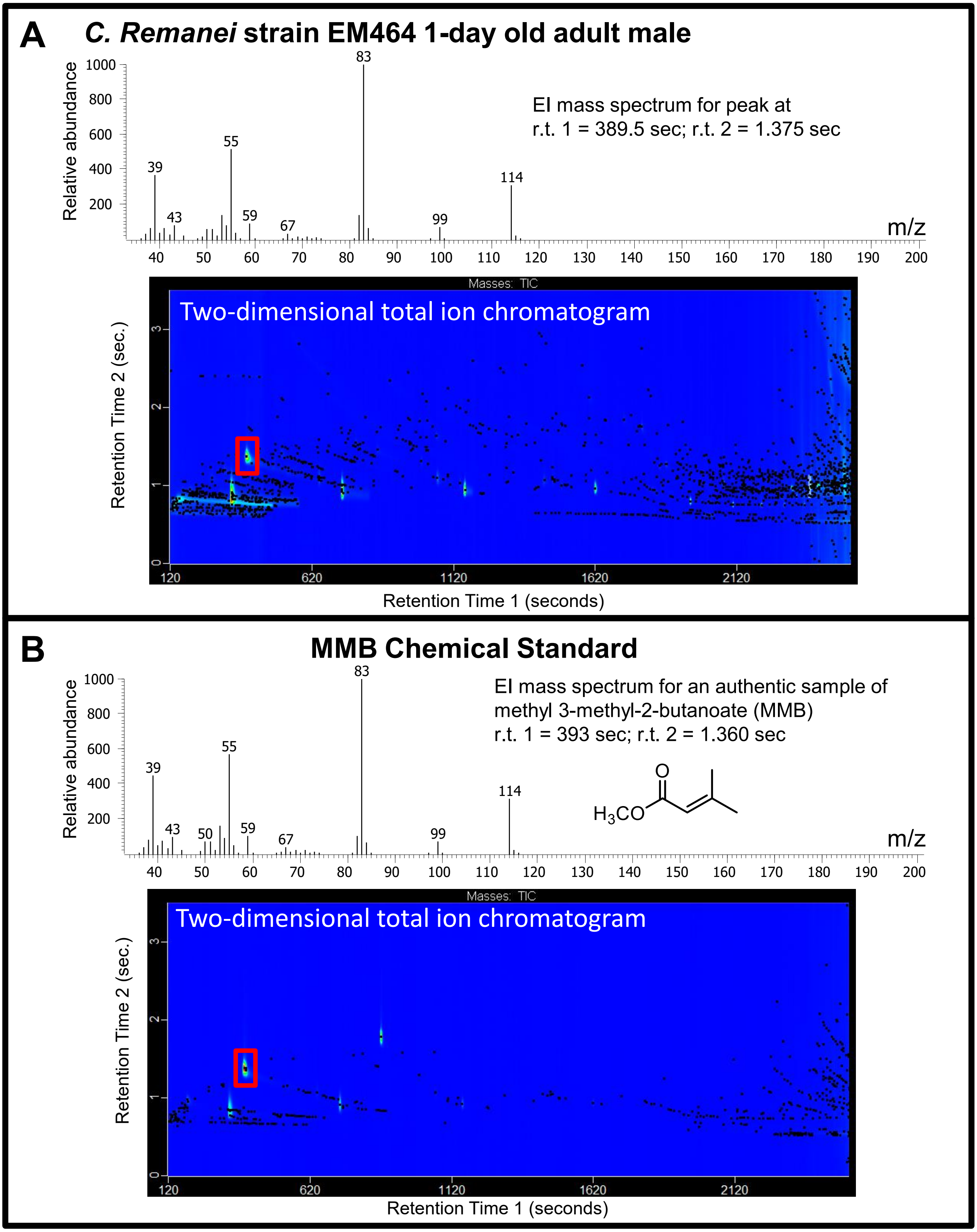


**Fig. S3: SPME headspace volatile sampling followed by GC x GC – TOFMS analyses revealed that MMB is produced by *C. remanei* adult males. (A)** EI mass spectrum and chromatogram (contour view) showing a chromatographic peak (indicated by red rectangle) which was unique to *C. remanei* adult male samples **(B)** Mass spectrum and chromatogram acquired for an authentic sample of MMB. Black dots represent discrete elution peaks identified during data processing (shown for a signal-to-noise threshold of 30).

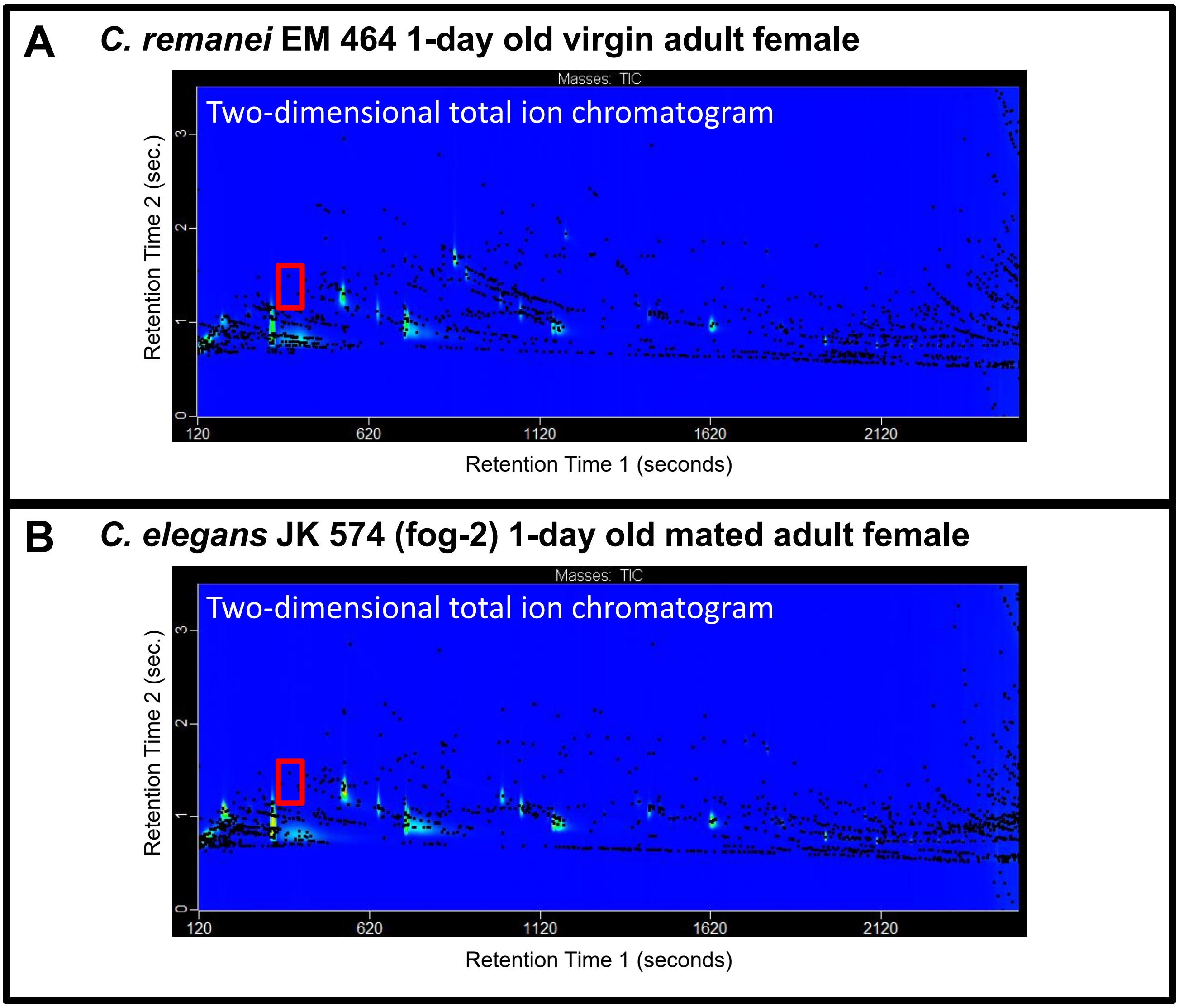


**Fig. S4** MMB was not detected for SMPE samples collected for *C. remanei* 1-day old virgin adult females, nor for any *C. elegans* sample. The red rectangle indicates the retention time region within which MMB would elute, if present.

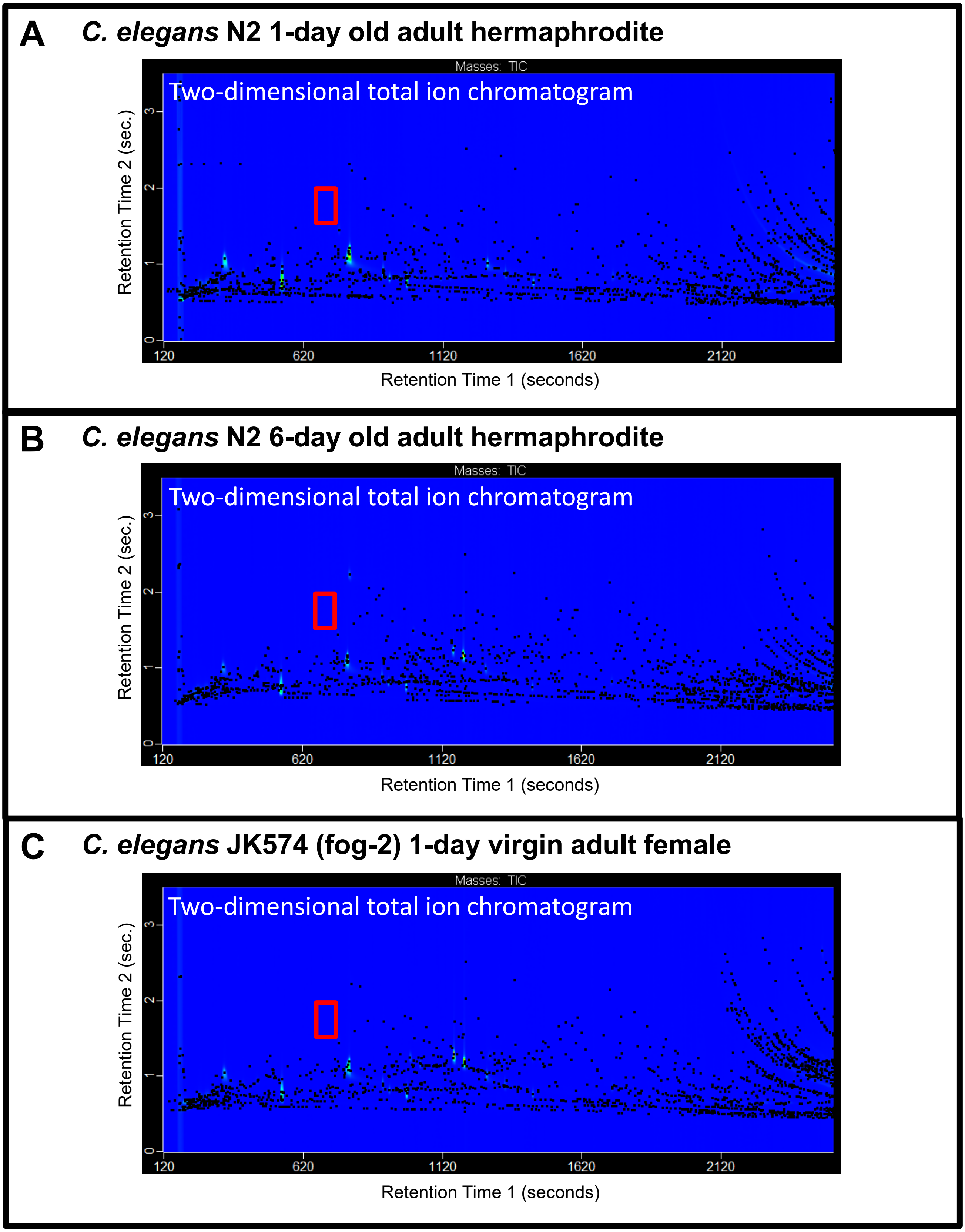


**Fig. S5** MMB was not detected for SMPE samples collected for any *C. elegans* sample. The chromatograms shown in this figure were acquired using an alternate secondary column (SGE Analytical Science BPX 50; described in Methods section), resulting in a change in the retention time region within which MMB would elute, if present (indicated by red rectangle).

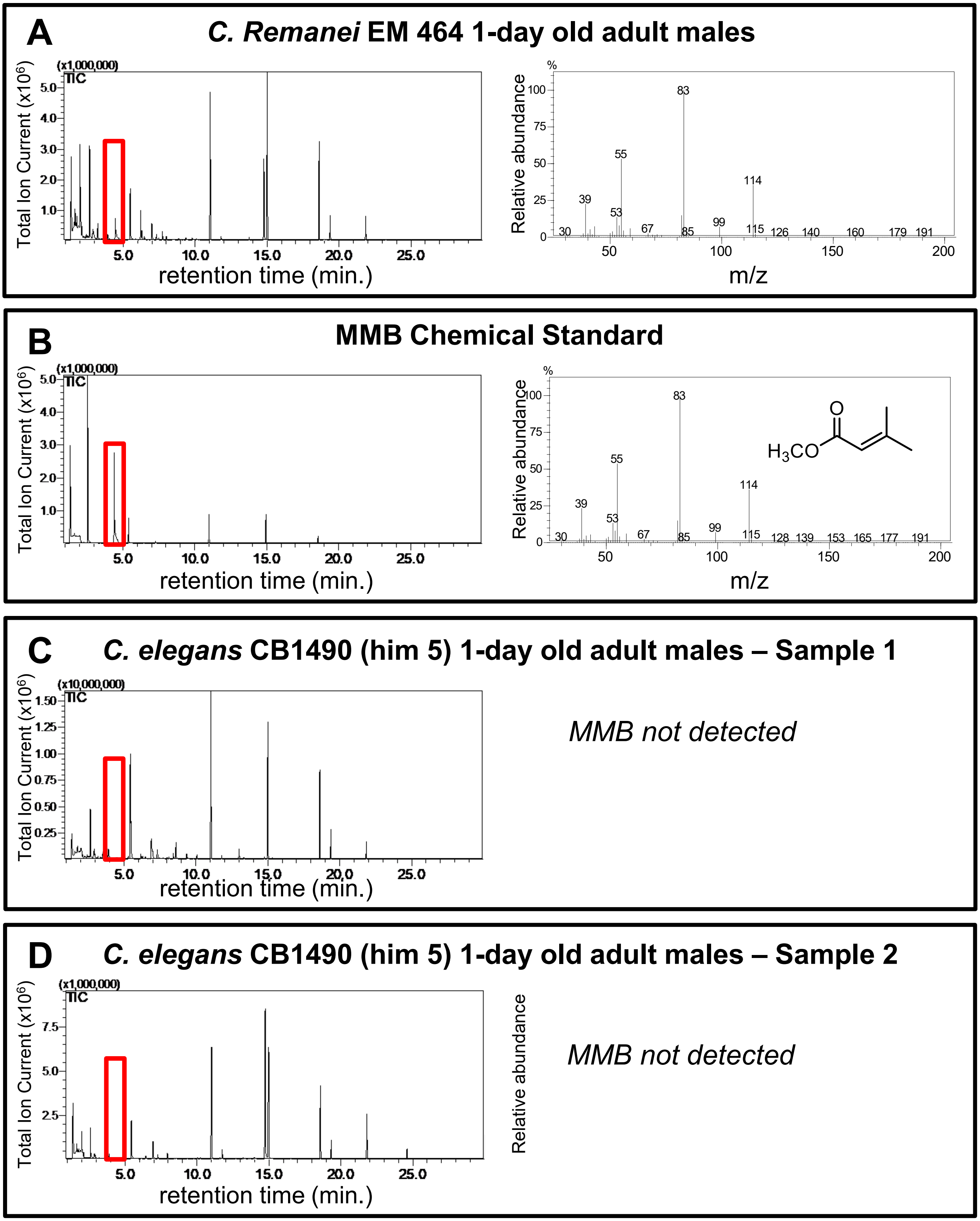


**Fig. S6 Additional analyses by SPME-GC-MS confirmed the presence of MMB for *C. remanei* adult male samples, as well as the absence of MMB for *C. elegans* adult males.** The red rectangle shows the retention time region within which MMB elutes, if present.
